# Supplementary material for: The interactome of intact mitochondria by cross-linking mass spectrometry provides evidence for coexisting respiratory supercomplexes
Source: Mol Cell Proteomics. 2017 Dec 8;17(2):216–32. doi: 10.1074/mcp.RA117.000470 (PMC5795388; doi:10.1074/mcp.RA117.000470)
Supplement: Supplemental Data [file supp_RA117.000470_133922_0_supp_23978_fzffwf.zip › spectra_annotation/mito_spectra_annotation/467-1-4-1-3-1.pdf]

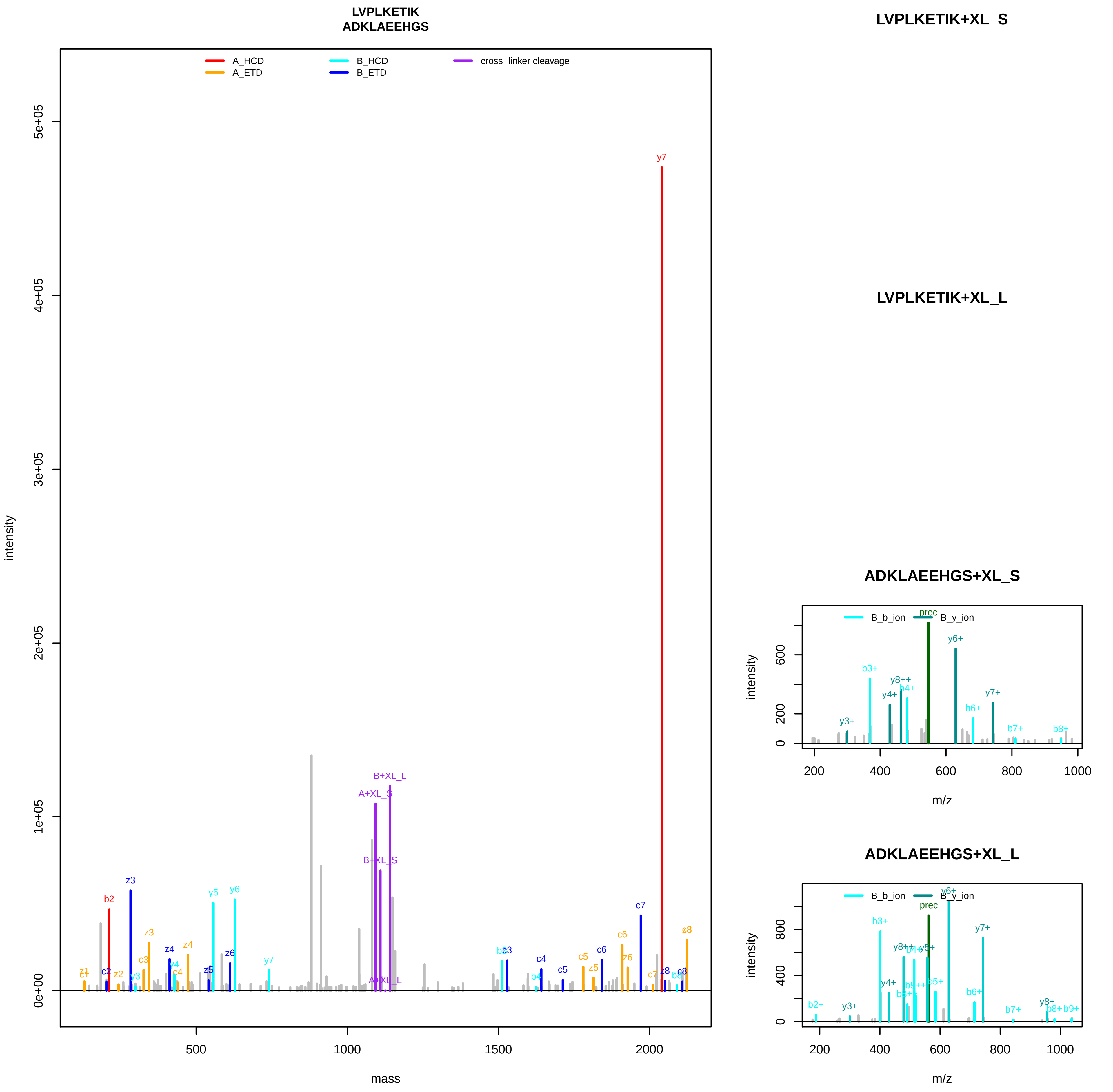

LVPLKETIK
ADKLAEEHGS
A_HCD
A_ETD
B_HCD
B_ETD
cross−linker cleavage
intensity
mass
y7
b2
z3
y5
y6
B+XL_L
A+XL_S
B+XL_S
A+XL_L
c7
c6
z8
c8
LVPLKETIK+XL_S
LVPLKETIK+XL_L
ADKLAEEHGS+XL_S
B_b_ion
B_y_ion
prec
y6+
b3+
y8++
y4+
b4+
y7+
b6+
y3+
b7+
b8+
m/z
ADKLAEEHGS+XL_L
b2+
b5+
b9+
y8+
